# Exploring bilateral field advantage across lifespan with a visual working memory span task: Experimental data, analysis and computer simulation

**DOI:** 10.1016/j.dib.2020.105502

**Published:** 2020-04-09

**Authors:** Anka Slana Ozimič, Grega Repovš

**Affiliations:** a Department of Psychology, University of Ljubljana, Aškerčeva 2, Ljubljana SI-1000, Slovenia; b Faculty of Medicine, University of Ljubljana, Vrazov trg 2, Ljubljana SI-1000, Slovenia

**Keywords:** Active maintenance, Bilateral field advantage, Healthy ageing, Orientations, Representational potential, Span task, Visual working memory capacity

## Abstract

This article describes the data collected in four experiments presented in the paper “Visual working memory capacity is limited by two systems that change across lifespan” [1]. The data includes behavioural results from a sample of 397 healthy participants performing a visual working memory span task in which they had to maintain the orientations of items presented to the left, right, or both visual hemifields. It also includes a simulation of experimental data for a number of possible scenarios. The repository [2] encompasses individual raw data files, a Python preprocessing script used for filtering raw data and the resulting dataset, an R script used to carry out the statistical analysis of the preprocessed data as well as an R script used for the simulations reported in the original paper. Finally, the repository includes an R generated analysis report, containing results of statistical tests and related visual materials, as well as the results of the simulation.

Specifications table

**Table:** 

Subject	Experimental and Cognitive Psychology
Specific subject area	Visual working memory and bilateral field advantage
Type of data	Individual log files Text file Data preprocessing script Analysis scripts and reports
How data were acquired	Experiments conducted on a computer with a custom script prepared in PsychoPy software [3]; Computer simulations prepared in R [4]
Data format	Raw Filtered Simulated Analysed
Parameters for data collection	Position of visual target items on the screen (up, down, left, right). Presence of spatial pre-cue indicating the position of target items.
Description of data collection	Human participants performed a visual working memory span task in which they had to maintain and reconstruct orientations of visual items.
Data source location	Faculty of Arts, University of Ljubljana, Ljubljana, Slovenia
Data accessibility	Repository name: Open Science Framework (OSF) Data identification number: DOI 10.17605/OSF.IO/GBUJW Direct URL to data: https://osf.io/gbujw/?view_only=82e09102e03b4634952699c683d56907[2]
Related research article	A. Slana Ozimič, G. Repovš, Visual working memory capacity is limited by two systems that change across lifespan, *Journal of Memory and Language.* 112 (2020). [1]

## Value of the data

• The data includes human performance in a novel visual working memory span task. It allows researchers to validate the published results, test alternative hypotheses and explore questions not addressed in the paper.
• Any researcher interested in working memory models and performance as well as researchers developing and testing new statistical models can benefit from using these data.
• Detailed individual reports of the acquired data are provided, which enables other researchers to extend the statistical analyses presented in the related paper and gives the opportunity for research collaboration.
• R code is provided to ensure reproducibility of the analysis of experimental data published in the related paper [1].
• Simulation data provides overview of model behaviour across a relevant parameter space and could be used for the development of further experiments.

## 1 Data description

All materials relevant for this paper are stored in a repository [2], which is organised into two main parts — *Experimental data,* and *Analyses and simulation. Experimental data* is described in the Data Description section, while the materials relevant for *Analyses and simulation* are presented under Experimental Design, Materials, and Methods section.

### 1.1 Raw data

The experimental data contains the folder *RawData* where raw data from 397 participants is stored as individual log files, produced by running a custom experimental script prepared in PsychoPy [3]. Each log file is named with a study code, followed by the experiment name and a subject specific code (e.g. *VWMST.exp1.UECS.txt*). The log file contains a 3-row header (marked with *#*), which includes subject code (row 1), demographic information (age, sex, handedness, status and years of education; row 2), and experimental conditions (row 3). The second part of the log file contains trial specific information. Each row begins with the description of the trial part (see Figure 2 in [1]): “ItiPre” (denotes first fixation), “Cue” (denotes pre-cue), “PreTarget” (denotes second fixation), “Targets” (denotes presentation of targets), “Delay” (denotes delay onset), “Trial” (denotes the end of trial). The numbers following the description of each trial part mark the timing of the start of that trial part. The “trial” part of the trial gives the most relevant information, as it contains information about the number of targets presented within a trial (working memory load) and the number of successfully recalled target orientations, as well as time needed to reconstruct them (e.g. …−2/3–7.56 would indicate that two out of three target orientations were correctly recalled and the time needed for the reconstruction was 7.56 s). The footer of the log file (marked with *#*) contains capacity estimates computed separately for each of the four target location conditions (down, right, up, left). Four capacity estimates were recorded and listed in the order as they are described in the Methods section.

### 1.2 Preprocessing script

The experimental data section in repository additionally contains a Python *DataPreprocessing.py* script, which is to be used for the preprocessing of the raw data. The script reads each individual log file and extracts the subject code, demographics, trial conditions and related capacity estimates into *PreprocessedData.txt* file.

### 1.3 Preprocessed data

*PreprocessedData.txt* file is a single file containing summarised data from all the subjects. Columns contain subject specific codes (*subject*), demographic data (*age, sex, hand, edu*), capacity estimates based on different equations (*V1-V4*; note that V1 was estimated based on Formula 1 described in the related paper [1]), information about presence of pre-cue (*cue*) and experiment number (*exp*). The file also specifies the target location (*side*) for the relevant capacity estimates (note that each participant's data is presented in four rows for four different locations: up, down, left, right). As an eye-tracker was used in Experiment 4, the file also contains data about fixation breaks (*fix_breaks* – the proportion of all fixation breaks, *tfix_breaks* – the proportion of fixation breaks on target presentation, *dfix_breaks* – the proportion of fixation breaks during delay, *tsfix_breaks* – the proportion of fixation breaks during target presentation computed for each target location).

### 1.4 Detailed raw data

In addition to the individual log files stored in the *RawData* folder, the experimental data part also contains a folder *RawDataDetailed* where detailed individual reports of the acquired data can be found. Detailed reports, in addition to information presented in basic log files, include information about the specific orientations of targets in each trial. In the main part of the report (between header and footer) this information can be found under the *trial* part of the trial. The 9th column marks the number of successfully recalled orientations, following by the reaction time and two orientation angle arrays. The first array provides orientations of all the presented stimuli, while the second array gives the orientations as reconstructed by the participants. The following array marks a match between the original and recalled orientation (*True* for a match in item orientation and *False* for a mismatch in item orientation). The last array lists the locations of each of the presented targets, where N corresponds to the position of that numeral on the clock shifted by 30 min (e.g. position 2 would equal the position of the small clock hand at 2:30).

## 2 Experimental design, materials, and methods

The data was collected in four separate experiments with slight variations in the experimental conditions and design. The rationale for the design and specific design changes are described in detail in the related research article [1]. Here we present the details of the task design and the specific differences between task conditions in the four experiments in which the data was collected.

### 2.1 Visual working memory span task

To estimate the capacity of visual working memory we designed a novel visual working memory span task. The task began with 12 circular grey placeholders (35 px in diameter) that were distributed equally on an imaginary circle 520 px in diameter on a white background with a small grey circle (20 px in diameter) placed in the centre. The first placeholder was positioned at an angle of 15° from the vertical, the rest followed with 30° separation. This ensured that there were three placeholders in each visual quadrant (upper left and right, and lower left and right) from the centre point defined by the small circle, and none directly on the vertical or horizontal lines from the centre. This visual setup persisted throughout the task in all four experiments. Each trial of the task started with the participant clicking on the central circle with a mouse. This enabled the task to be self-paced and also ensured that the participant's gaze was fixed at the centre of the screen at the start of the trial. Upon the click, the centre circle changed colour from light grey to dark grey for the duration of the trial. A 500  ms delay followed after trial start. When the task included a pre-cue, then after the initial delay the placeholders where the target items were to be shown next turned light grey for 50  ms, followed by another 500 ms delay. Next, targets were shown for 500 ms. The targets were black *keys* (35 px in length), composed of a small circle (19 px in diameter) and a line (7 px wide) pointing from the circle in one of eight possible directions: 0°, 45°, 90°, 135°, 180°, 225°, 270°, and 315°. Depending on the condition, the targets were shown either on the top, left, right, or bottom side of the imaginary circle replacing the relevant placeholders. When the number of targets was even, they were shown in equal numbers to the left and right of the vertical midpoint (for the top and bottom presentation) and above and below the horizontal midpoint (for the left and right presentation). When the number of targets was odd, the odd target was presented randomly either immediately clockwise or anticlockwise from the main group. The orientations of the targets in the target set where chosen randomly from the eight possible orientations without replacement. The task of the participant was to remember the orientations of the targets shown. Target presentation was followed by a 2 s delay in which the placeholders where shown again. After the delay, the response period started. On the response screen, the placeholder at each of the original target positions was replaced by a simplified eight-point compass rose (35 px in diameter). The participants were asked to recreate the orientations of the targets shown previously by clicking on the relevant hand of the compass rose with a mouse pointer, at which time the compass rose changed to a key pointing in the indicated direction. The participants were free to provide responses in any order, as well as to change the provided responses. When the participants were satisfied with their reconstruction of the original target display, they submitted the reconstruction by clicking on the central fixation point. At that time feedback was given by changing the colour of the fixation point to either green or red for 500  ms, for correct and incorrect reconstructions, respectively. Finally, the colour of the fixation point returned to light grey, indicating the rest period before the onset of a new trial. Importantly, the participants were instructed to maintain their gaze on the middle fixation point throughout the trial, except for the target reconstruction in the response period.

### 2.2 Capacity estimation procedure

Within each task run the visual working memory capacity was estimated for each of the four presentation locations (left, right, top, bottom) using the following span procedure. The procedure started with one target item. If the item was correctly reconstructed in the first trial, the load in the next trial with the target presentation at the same location increased by one. If the reconstruction was incorrect, the participant was given up to four more opportunities at that load and could progress to an increased load if at least three trials were completed successfully. If no trials were completed successfully at a given load, the load was reduced by one until the participant completed five trials at the highest load with at least one successful trial. The span for that condition was computed in the following ways. First as the maximum achieved load with at least one correct response (*N*) corrected by the proportion of correct responses at that load:

(1) $$K=N-1+\;\frac{C_{N}}{5}$$

Where *K* is the estimated capacity, *N* is the highest load with at least one correct response, and *C_N_* is the number of correct responses at load *N*.

Second, as the average number of correctly reconstructed items at load *N*:

(2) $$K=\;\frac{\sum_{i=1}^{5}C_{Ni}}{5}$$

Where *K* is the estimated capacity *i* is the trial number at load N and *C_Ni_* is the number of correctly reconstructed items at trial *i* at load *N*.

Third, as the maximum achieved load with at least one correct response (*N*).

Fourth, as the maximum load at which at least three correct responses were given (*N_3_*).

The trials for each of the four target presentation location conditions were intermixed in a random order. The task run ended when the span procedure completed for all four target presentation location conditions. For each trial we recorded the specific target orientations, responses provided and total reaction time for the trial.

### 2.3 Task conditions across experiments

Besides target presentation location, which—as described above—was manipulated within each task run, the following conditions differed or were manipulated across the four experiments. In experiments 1 and 2, the task was performed using participants’ or experimenters’ computers, the distance from the screen was not explicitly controlled, and in both experiments spatial pre-cues were used in all trials. In experiment 3, the data collection for all the participants was performed using the same computer setup (Mac Mini with an Intel Core Duo 2.0 G Hz CPU and 8 GB RAM, running Mac OS 10.12, and ASUS VG248QE 24″ screen using the native resolution of 1930 × 1080 px and a 60 Hz refresh rate). The participants were seated 55  cm from the screen, which ensured constant stimuli sizes. Specifically (all measures given in degrees of visual angle), target stimuli were 1.08° long with the main circle 0.59° in diameter and the line 0.22° wide, placeholders and probe stimuli were 1.08° in diameter, the fixation point was 0.62° in diameter, the invisible circle on which the placeholders, targets and probe stimuli were placed was 15.65° in diameter. No spatial pre-cue was shown in experiment 3. In experiment 4, the data collection for all the participants was again performed using the same equipment (in this case a Mac Mini computer with a 3.6  GHz quad-core Intel Core i3 processor and 8 GB RAM running the 64-bit version of Windows 10, 24″ BenQ XL2420G screen using the native resolution of 1920 × 1080 px and a 60 Hz refresh rate, EyeLink 1000 eye-tracking system with single-eye tracking at a 1000 Hz sample rate). The participants were seated 70  cm from the screen with their head placed on a fixed chin rest, again ensuring fixed stimuli sizes. Specifically (all measures given in degrees of visual angle), target stimuli were 0.78° long with the main circle 0.42° in diameter and the line 0.16° wide, placeholders and probe stimuli were 0.78° in diameter, the fixation point was 0.44° in diameter, the invisible circle on which the placeholders, targets and probe stimuli were placed was 11.38° in diameter. Participants in experiment 4 completed 2 task runs, one with and one without spatial pre-cues. The order of the runs was randomly changed across participants. Additionally, if the participant's gaze during target presentation and delay periods deviated from the fixation point more than 100 px (2.22° visual angle), the trial was aborted and did not count towards computation of the span.

### 2.4 Analysis and simulation

The *Analyses and simulation* component of the described repository [2], which contains the materials used for performing statistical analyses and computer simulation, contains two folders. The *Experimental data analysis* folder provides an R script (*Analysis_V1.R*) that was used for the analysis presented in the related research article [1], as well as the related analysis report (*Analysis_V1.html*), which contains both R code commands as well as their results. The report was prepared in R [4] using the *knitr* [5] package. Note that *Analysis_V1.R* script takes PreprocessedData.txt as the input data file. The *Simulation* folder contains an R script used to perform the simulation (*simulation.R*) and the compiled report file (*simulation.html*) presented in the supplementary material of the related research paper [1].
